# Genome-Wide Analysis of the GRF Family Reveals Their Involvement in Abiotic Stress Response in Cassava

**DOI:** 10.3390/genes9020110

**Published:** 2018-02-20

**Authors:** Sang Shang, Chunlai Wu, Chao Huang, Weiwei Tie, Yan Yan, Zehong Ding, Zhiqiang Xia, Wenquan Wang, Ming Peng, Libo Tian, Wei Hu

**Affiliations:** 1 Tropical Agriculture and Foresty Institute, Hainan University, Haikou 570228, Hainan, China; fair.play@163.com; 2 Key Laboratory of Biology and Genetic Resources of Tropical Crops, Institute of Tropical Bioscience and Biotechnology, Chinese Academy of Tropical Agricultural Sciences, Haikou 571101, Hainan, China; wuchunlai19900109@126.com (C.W.); tieweiwei@itbb.org.cn (W.T.); yoyoyan7758@163.com (Y.Y.); dingzehong@itbb.org.cn (Z.D.); xiazhiqiang@itbb.org.cn (Z.X.); wangwenquan@itbb.org.cn (W.W.); pengming@itbb.org.cn (M.P.); 3 College of Life Science and Technology, Huazhong University of Science and Technology (HUST), Wuhan 430074, Hubei, China; huangchao2005@hust.edu.cn

**Keywords:** cassava, abiotic stress, gene expression, identification, GENERAL REGULATORY FACTOR (GRF) protein family

## Abstract

GENERAL REGULATORY FACTOR (GRF) proteins play vital roles in the regulation of plant growth, development, and response to abiotic stress. However, little information is known for this gene family in cassava (*Manihot esculenta*). In this study, 15 MeGRFs were identified from the cassava genome and were clustered into the ε and the non-ε groups according to phylogenetic, conserved motif, and gene structure analyses. Transcriptomic analyses showed eleven *MeGRFs* with constitutively high expression in stems, leaves, and storage roots of two cassava genotypes. Expression analyses revealed that the majority of *GRFs* showed transcriptional changes under cold, osmotic, salt, abscisic acid (ABA), and H_2_O_2_ treatments. Six *MeGRFs* were found to be commonly upregulated by abiotic stress, ABA, and H_2_O_2_ treatments, which may be the converging points of multiple signaling pathways. Interaction network analysis identified 18 possible interactors of MeGRFs. Taken together, this study elucidates the transcriptional control of *MeGRFs* in tissue development and the responses of abiotic stress and related signaling in cassava. Some constitutively expressed, tissue-specific, and abiotic stress-responsive candidate *MeGRF* genes were identified for the further genetic improvement of crops.

## 1. Introduction

GENERAL REGULATORY FACTOR (GRF) proteins are also named 14–3–3 proteins that are widely distributed in eukaryotes. They are characterized as the most important phosphopeptide-binding proteins, playing a regulatory role in various biological processes [1,2,3,4]. Through the specific groove structures formed by their homo- or hetero-dimers, GRF proteins function on regulating various phosphorylated clients, including metabolic enzymes, transporters, protein kinases, and transcription factors [2,4,5,6,7]. Based on the interaction of GRFs with different clients, many biochemical changes occurred, including intracellular localization, activity, degradation, stability, and the binding ability with other client proteins [2,3,4,5].

In 1992, the first plant 14–3–3 protein was characterized from maize [8]. Then, 14–3–3 proteins were identified in various species, including eight from rice (*Oryza sativa*) [9,10], 13 from *Arabidopsis thaliana* [2,11,12], 18 from soybean (*Glycine soja*) [13], 31 from cotton (*Gossypium hirsutum*) [14], nine from common bean [15], twelve from tomato [16], 14 from *Populus trichocarpa* [17], 21 from *Brassica rapa* [18], eight from *Brachypodium distachyon* [19], and 25 from banana [6]. Further studies demonstrated that GRF proteins played a crucial role in the regulation of plant growth and development in various species. Interference of ε group 14–3–3 genes altered auxin distribution and led to agravitropic growth in *Arabidopsis* [20]. Multiple mutant analyses revealed the regulatory role of 14–3–3 proteins in chloroplast division, root growth, leaf longevity, and photosynthesis [21,22,23]. GsGF14o was involved in stomatal and root hair development in *G. soja* [24]. Gh14–3–3L functioned on fiber elongation and maturation in cotton [25]. Additionally, numerous evidences revealed that GRF proteins were involved in abiotic stress response. OsCPK21 could phosphorylate OsGF14e to enhance plant response to salt stress and abscisic acid (ABA) [26]. AtGCN4 function on degrading RIN4 and 14–3–3 proteins, promoting stomatal closure, and increasing drought tolerance [27]. *BdGF* genes showed induction after abiotic stresses or hormone treatments in *B. distachyon* and overexpression of BdGF14a improved plant tolerance to drought stress [28]. Overexpression of *BdGF14d* increased plant tolerance to salt stress by regulating ABA pathway, reactive oxygen species (ROS) scavenging system, and ions transports [19]. CRPK1 phosphorylated 14–3–3 proteins, leading to 14–3–3 proteins transfer from cytosol to nucleus, destabilization of CBF proteins, and regulation of cold response in *Arabidopsis* [29]. Together, these studies suggest that 14–3–3 proteins play a regulatory role during plant growth, development, and in responses to stresses.

Cassava is the sixth most important crop after wheat, rice, maize, potato, and barley [30]. Its edible tuberous roots supply source of dietary carbohydrate for over 600 million people worldwide [31]. Cassava is also considered as a potential biofuel crop for production of ethanol and bioenergy due to its high starch production [30]. Cassava also shows high resistance to abiotic stress, such as drought and low nitrogen [32]. However, the mechanism underlying cassava resistance to abiotic stress is largely unknown. Although 14–3–3 proteins have been confirmed to play an important role in plant response to abiotic stress in various species, less information is known concerning this gene family in cassava. In this study, a total of 15 cassava GRFs were characterized. Their phylogenetic relationships, protein motifs, gene structure, expression profiles in various tissues and in response to abiotic stress, and interaction network were systematically analyzed. This comprehensive analysis should further our understanding of the GRF functions associated with abiotic stress response, as well as inform the genetic improvement of crops.

## 2. Materials and Methods

### 2.1. Plant Materials and Treatments

W14, (*Manihot esculenta* ssp. *flabellifolia*), originally collected in Brazil, is the nearest ancestor of cultivated cassava, and has the characteristics of low photosynthesis rate, tuber root yield, and starch content in root tubers. Arg7 (*M. esculenta* Crantz cv. Arg7), adapted to geographical high-latitude region of Argentina, is a variety containing elite agronomic traits, including a certain level of growth under moderate drought stress. W14 and Arg7 were cultured in pots with a mixture of soil and vermiculite (1:1) under a growth room with a 16 h/35 °C day and 8 h/20 °C night regime, and a relative humidity of 70%. Stems (90 days after planting), leaves (90 days after planting), and storage roots (150 days after planting) in Arg7 and W14 were collected to study the transcriptional changes of cassava genes in different organs under normal growth conditions. For osmotic, cold, salt, ABA, and H_2_O_2_ treatments, Arg7 variety was cultured with Hoagland solution (Tuopu, Qingdao, China) under normal growth conditions. Sixty-days-old seedlings of Arg7 were subjected to aqueous solutions of 200 mM mannitol for 14 d, 300 mM NaCl for 14 d, 100 µM ABA for 24 h, 10% H_2_O_2_ for 24 h or low temperature (4 °C) for 48 h, respectively. Then, the leaves were sampled to perform quantitative real-time polymerase chain reaction (qRT-PCR) analysis.

### 2.2. Identification and Evolutionary Analysis

The GRF protein sequences in *Arabidopsis* and rice were acquired from UniProt and rice genome annotation project (RGAP), respectively [33,34]. The genome sequences of cassava were downloaded from the cassava genome database [35]. The known GRF sequences were used to build hidden markov models (HMM) for searching the cassava dataset using HMMER software [36,37]. The identified cassava GRFs were further validated by basic local alignment search tool (BLAST) analysis using GRFs from rice and *Arabidopsis* as queries [38]. Based on the conserved domains database (CDD) database, the conserved domain of cassava GRFs were validated [39,40]. To study the evolutionary relationship of cassava GRFs, MEGA 5.0 and Clustal X 2.0 softwares were employed to construct the evolutionary tree with the entire protein sequences of GRFs from cassava, *Arabidopsis*, and rice [41,42]. The protein sequences of cassava GRFs were also used to construct evolutionary tree and protein motif analysis.

### 2.3. Transcriptome Analysis

Total RNA of stems, leaves, and storage roots in Arg7 and W14 was extracted with plant RNA extraction kit (Tiangen, Beijing, China) for complementary DNA (cDNA) library construction. The sequencing was performed with an Illumina GAII (Illumina, San Diego, CA, USA) following manufacturer’s instructions. Adapter sequences were removed with FASTX-toolkit [43]. Clean reads were generated by removing low quality sequences using FastQC [44]. Tophat v.2.0.10 was used to map the clean reads to the cassava genome [45]. Using cufflinks, the transcriptome data was assembled [46]. Fragments per kilobase of transcript per million fragments mapped (FPKM) was employed to calculate gene expression levels. Each sample has three replicates.

### 2.4. Sequence Analysis

Using the ExPASy proteomics server, the molecular weight (MW) and isoelectric points (pI) of cassava GRFs were predicted [47]. The conserved motifs of cassava GRFs were identified with MEME and InterProScan databases [48,49]. The gene structures of cassava GRFs were assessed with the gene structure display server (GSDS) database [50]. Using STRING [51] with option value >0.7, specific interaction network with experimental evidences of GRFs in *Arabidopsis* were constructed, which identifies 18 high confidence interactive proteins in *Arabidopsis*. Then, the homologs of these interactive proteins in cassava were identified with reciprocal best BLASTP analysis.

### 2.5. qRT-PCR Analysis

The relative expression levels of cassava *GRFs* were examined by qRT-PCR analysis using StratageneMx3000P (Stratagene, CA, USA) instrument and SYBR Premix Ex Taq (TaKaRa, Dalian, China). The 2^−ΔΔCt^ method was used to assess the relative expression of cassava *GRF* genes [52]. The appropriate primer pairs were selected by melting curve, agarose gel electrophoresis, and sequencing PCR products (Table S1). The amplification efficiency was in the range of 0.91–1.07. The relative expression of cassava *GRF* genes in each time point was calculated according to the control and treated samples. Each sample has three replicates and three biological experiments were performed.

## 3. Results

### 3.1. Identification and Phylogenetic Analysis of GRF Gene Family in Cassava

Both BLAST and Hidden Markov Model searches were employed to extensively identify cassava GRF genes with GRF sequences from *Arabidopsis* and rice as query. As a result, 15 predicted full-length MeGRFs (MeGRF1–MeGRF15) were identified from cassava and their sequences were shown in Table S2. The cassava MeGRF proteins ranged from 232 to 264 amino acid residues in length, and their relative molecular mass varied from 26.25 kDa to 30.06 kDa, with the pIs in the range of 4.72 to 5.03 (Table S2).

**Figure 1:**
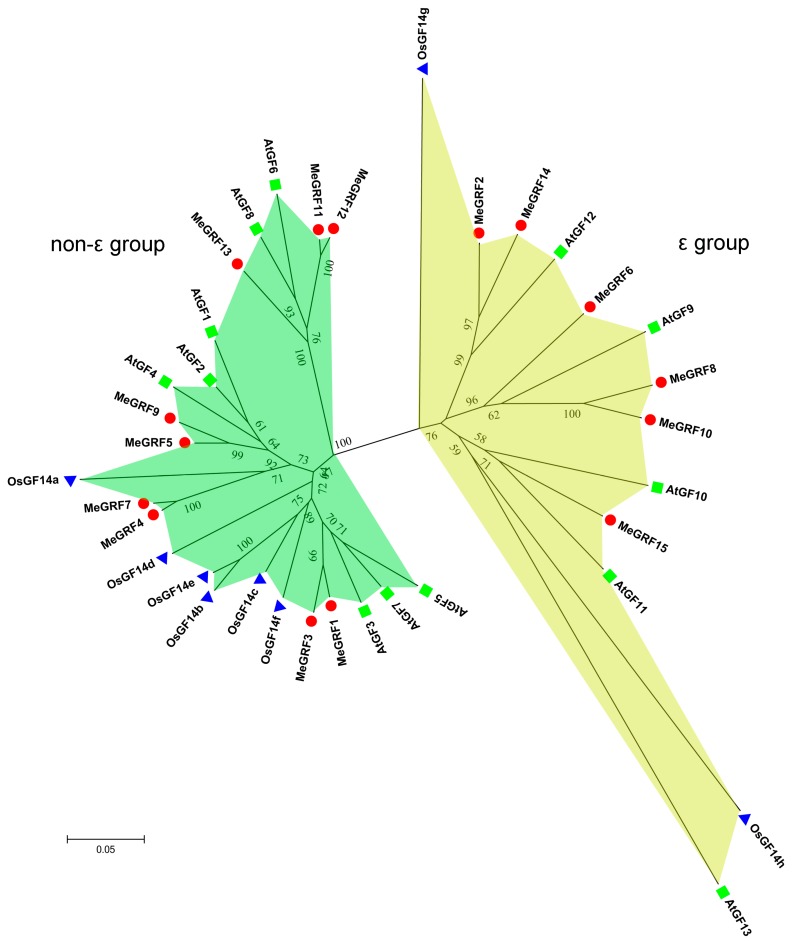
Phylogenetic analysis of the GENERAL REGULATORY FACTOR proteins (GRFs) from *Arabidopsis*, rice, and cassava. The neighbor-joining phylogenetic tree was constructed using ClustalX 2.0 and MEGA5.0 with 1000 bootstraps replicates [41,42].

A phylogenetic tree was constructed to understand the evolutionary relationships among GRF proteins from cassava, rice, and *Arabidopsis* (Figure 1; Table S3). The results suggested that all the GRFs were clustered into the ε group and the non-ε group. The ε group included MeGRF-2, -6, -8, -10, -14, -15, AtGF-9, -10, -11, 12, -13, and OsGF14-g, -h. The non-ε group contained MeGRF-1, -3, -4, -5, -7, -9, -11, -12, -13, AtGF-1, -2, -3, -4, -5, -6, -7, -8, and OsGF14-a, -b, -c, -d, -e, -f. Generally, GRFs from cassava showed closer relationship with GRFs from *Arabidopsis* than those from rice, which is consistent with the current understanding of plant evolutionary relationship.

### 3.2. Conserved Motif and Gene Structure Analyses of GRF Gene Family in Cassava

**Figure 2:**
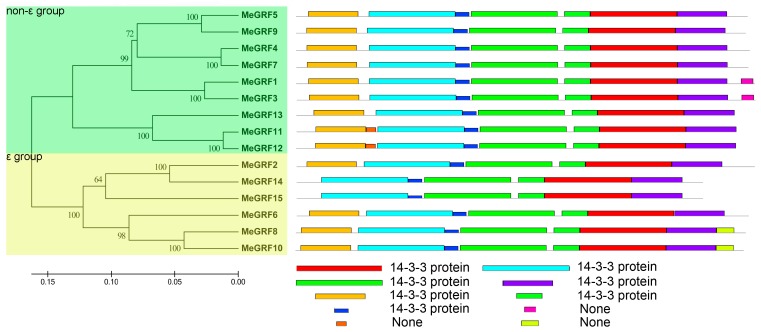
Phylogenetic and motif analyses of cassava GRFs. All cassava GRF protein sequences were determined with MEME software and were classed into two groups based on phylogenetic relationship [48].

To better understand the structural divergence and prediction the function of MeGRF proteins, a total of ten conserved motifs of cassava GRFs were found using MEME and further annotated by InterPro Scan 5 [48,49] (Figure 2). Seven motifs (motifs 1, 2, 3, 4, 6, 7, 8) were annotated as 14–3–3 protein motifs, which are the basic characteristics of the GRF family. Based on the motif assay, all the identified cassava GRFs showed at least six 14–3–3 protein motifs, indicating their typical characteristic of GRF family. Interestingly, some close homologous GRFs exhibited the same motif organization, including MeGRF1/3 specially having motif 9, MeGRF11/12 uniquely showing motif 5, and MeGRF8/10 uniquely containing motif 10, which further supports the phylogenetic classification of GRF family.

**Figure 3:**
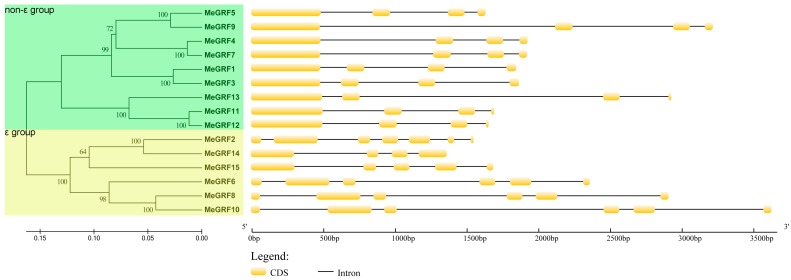
Exon-intron structure analyses of cassava GRFs. Gene structure display server (GSDS) database was used to analyze the exon-intron structures of MeGRFs. The yellow boxes represent exons and the black lines are introns [50].

Additionally, exon-intron organization among the coding sequence was examined to understand the structural diversity and evolution of cassava GRF family. As shown in Figure 3, it is clear that the ε group contained 4–7 exons, whereas the non-ε group showed 4 exons. The distinct exon-intron organization between the ε group and the non-ε group indicated their diversity during evolution, further supporting the phylogenetic classification of cassava GRFs.

### 3.3. Expression Profiles of MeGRF Genes in Different Tissues

**Figure 4:**
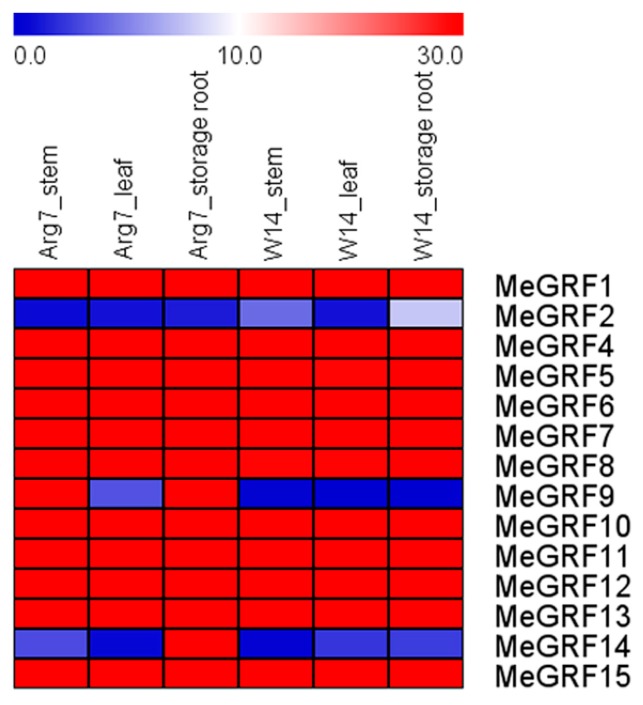
Expression analysis of cassava GRFs in stems, leaves, and storage roots of W14 and Arg7. The heat-map was created according to the Fragments per kilobase of transcript per million fragments mapped (FPKM) value of *MeGRF*s. Changes in gene expression are shown in color as the scale.

To study the expression profiles of *MeGRF* genes in different tissues, transcriptomic analyses were carried out from samples of leaves, stems, and storage roots in a wild subspecies (W14) and cultivated variety Arg7 (Figure 4; Table S4). The results revealed that 11 GRFs (*MeGRF-1*, *-4*, *-5*, *-6*, *-7*, *-8*, *10*, *-11*, *-12*, *-13*, *-15*) showed constitutively high expression (FPKM > 29) in all the tested tissues of the two genotypes. Interestingly, *MeGRF2* showed tissue specific expression pattern, with transcripts lacking in leaves and low expression (FPKM < 10) in stems and storage roots of the two genotypes. *MeGRF9* showed high expression in stems and storage roots of Arg7, but low expression in leaves of Arg7 and in all the tested tissues of W14. *MeGRF14* had high expression in storage roots of Arg7, whereas low expression in other tissues tested. These results implied the same or differential roles of cassava *GRF* genes in tissue development.

### 3.4. Differential Expression of MeGRF Genes in Response to Cold, Osmotic, Salt, ABA, and H_2_O_2_ Treatments

**Figure 5:**
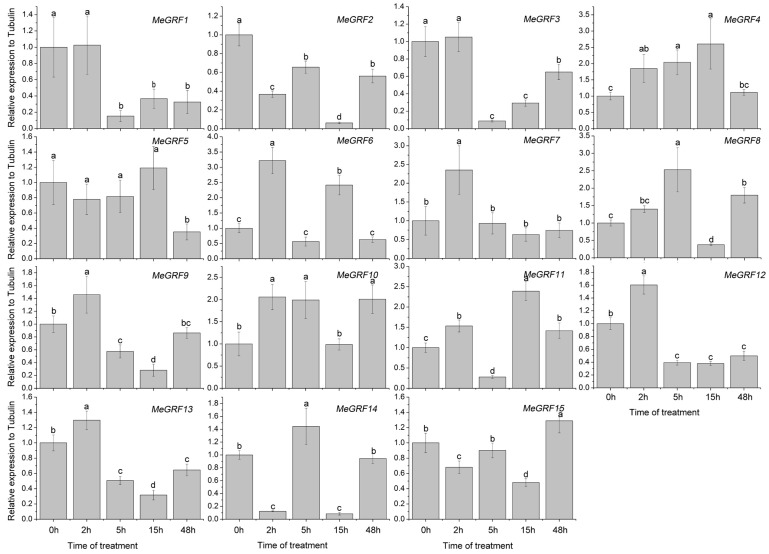
Expression analysis of cassava GRFs in response to cold treatment. The mean fold changes of each gene between treated and control samples at each time point were used to calculate its relative expression levels. No treatment control (NTC) indicates no treatment controls (mean value = 1). Data are means ± standard deviation, SD of *n* = 3 biological experiments. Means denoted by the same letter do not significantly differ at *p* < 0.05 as determined by Duncan’s multiple range test.

**Figure 6:**
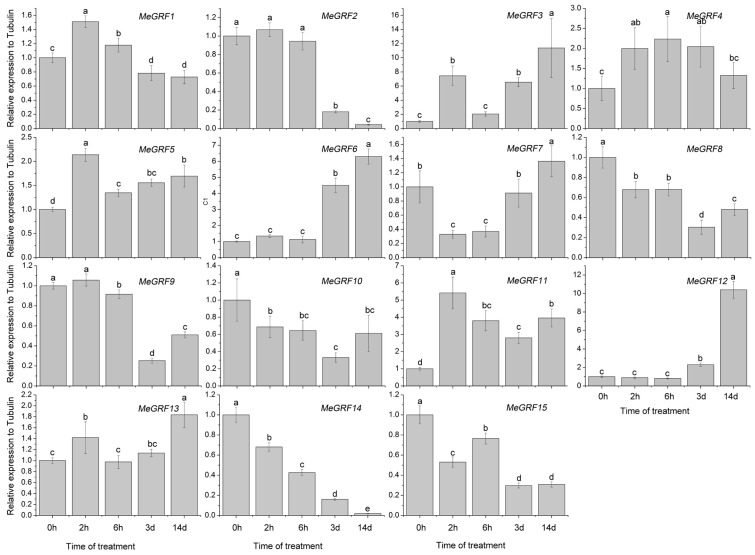
Expression analysis of cassava GRFs in response to salt treatment. The mean fold changes of each gene between treated and control samples at each time point were used to calculate its relative expression levels. NTC indicates no treatment controls (mean value = 1). Data are means ± SD of *n* = 3 biological experiments. Means denoted by the same letter do not significantly differ at *p* < 0.05 as determined by Duncan’s multiple range test.

**Figure 7:**
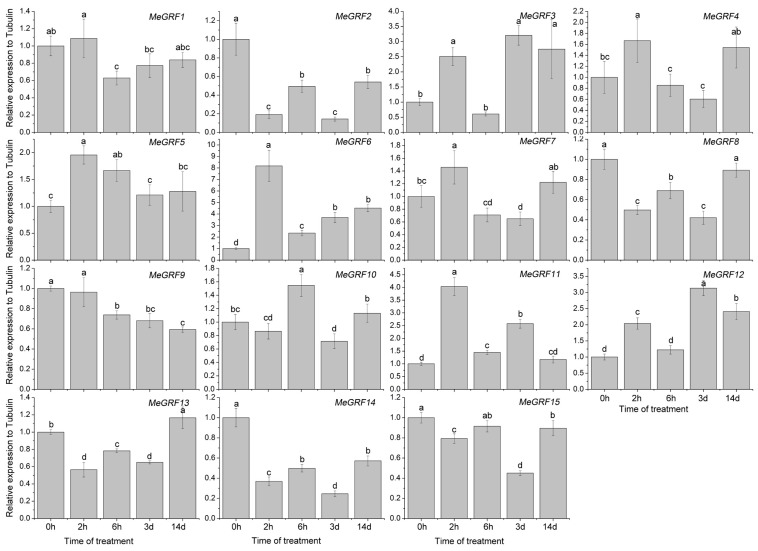
Expression analysis of cassava GRFs in response to osmotic treatment. The mean fold changes of each gene between treated and control samples at each time point were used to calculate its relative expression levels. NTC indicates no treatment controls (mean value = 1). Data are means ± SD of *n* = 3 biological experiments. Means denoted by the same letter do not significantly differ at *p* < 0.05 as determined by Duncan’s multiple range test.

**Figure 8:**
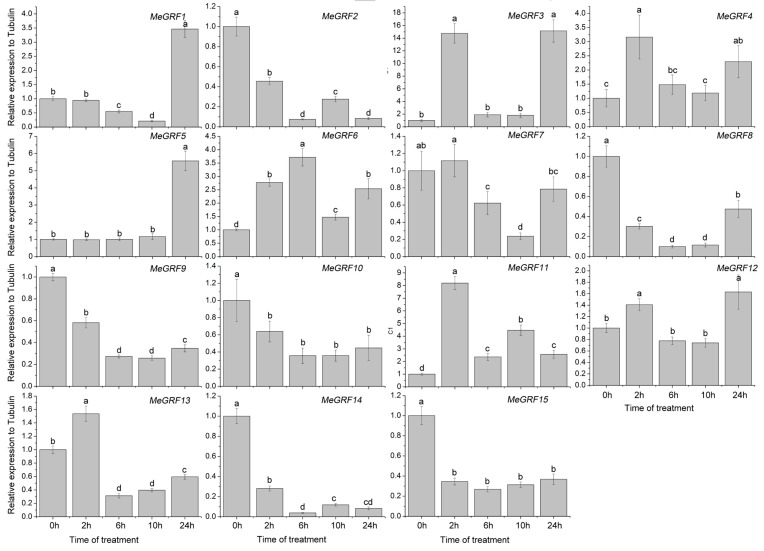
Expression analysis of cassava GRFs in response to ABA treatment. The mean fold changes of each gene between treated and control samples at each time point were used to calculate its relative expression levels. NTC indicates no treatment controls (mean value = 1). Data are means ± SD of *n* = 3 biological experiments. Means denoted by the same letter do not significantly differ at *p* < 0.05 as determined by Duncan’s multiple range test.

**Figure 9:**
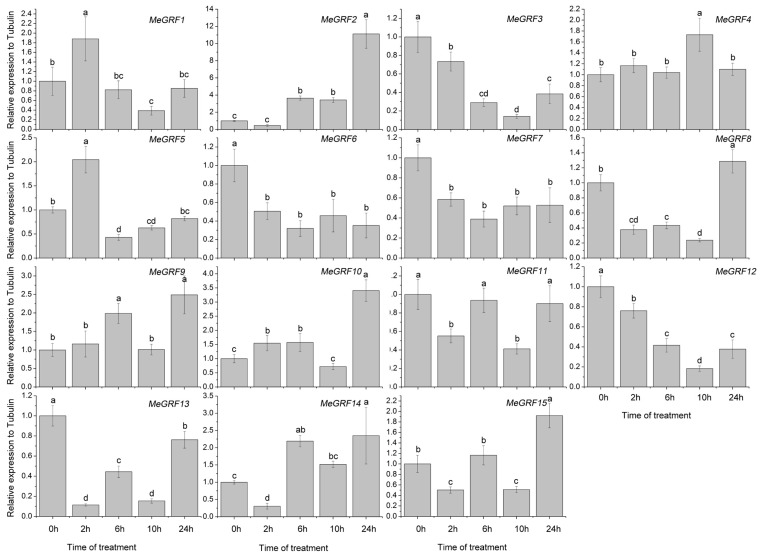
Expression analysis of cassava GRFs in response to H_2_O_2_ treatment. The mean fold changes of each gene between treated and control samples at each time point were used to calculate its relative expression levels. NTC indicates no treatment controls (mean value = 1). Data are means ± SD of *n* = 3 biological experiments. Means denoted by the same letter do not significantly differ at *p* < 0.05 as determined by Duncan’s multiple range test.

To investigate the transcriptional responses of *MeGRF* genes to abiotic stress and related signaling, cassava seedlings were subjected to cold, osmotic, salt, ABA, and H_2_O_2_ treatments (Figure 5, Figure 6, Figure 7, Figure 8 and Figure 9). Under cold treatment, *MeGRF-4*, *-6*, *-7*, and *-10* showed induction, whereas *MeGRF-1*, *-2*, *-3*, and *-5* showed repression. Under salt treatment, *MeGRF-3*, *-4*, *-5*, *-6*, *-11*, *-12*, and *-13* were upregulated, while *MeGRF-2*, *-8*, *-9*, *-10*, *-14*, and *-15* were downregulated. Under osmotic treatment, *MeGRF-3*, *-5*, *-6*, *-10*, *-11*, and *-12* showed upregulation, whereas *MeGRF-1*, *-2*, *-8*, *-9*, *-14*, and *-15* showed downregulation. Under ABA treatment, *MeGRF-3*, *-4*, *-5*, *-6*, *-11*, and *-12* transcripts increased, whereas the transcripts of *MeGRF-2*, *-7*, *-8*, *-9*, *-10*, *-14*, and *-15* decreased. Under H_2_O_2_ treatment, *MeGRF-2*, *-4, -9*, and *-10* were induced, whereas *MeGRF-3*, *-6*, *-7*, *-11*, *-12*, and *-13* were repressed. These results indicated that most of the *MeGRF* genes showed transcriptional changes under abiotic stress and related signaling molecule treatments.

### 3.5. Analysis of GRF Family Interaction Network

**Figure 10:**
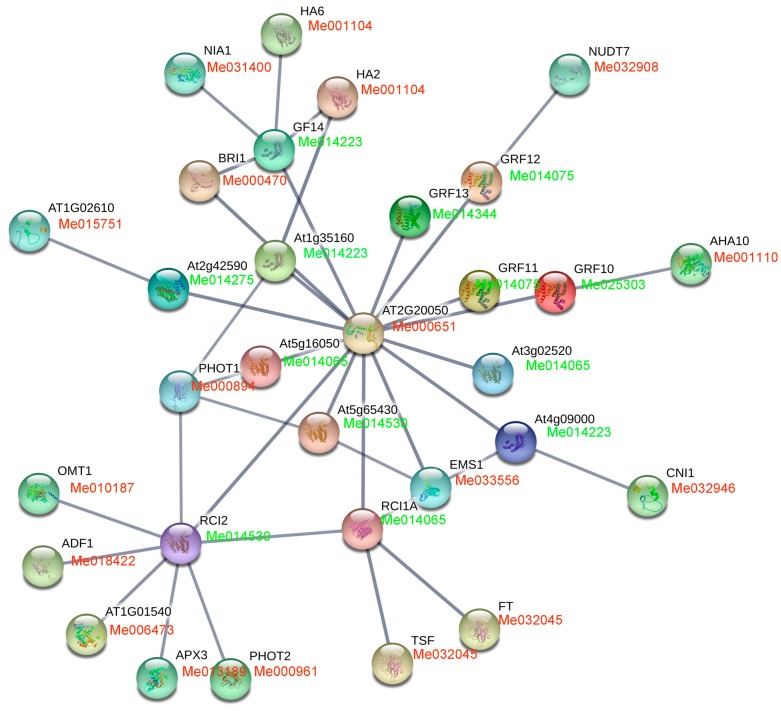
Interaction network analyses of GRFs in *Arabidopsis* and cassava. The upper one in the nodes represent genes in *Arabidopsis*. The lower one in the nodes represent genes in cassava. The genes marked with green font show GRFs; the genes marked with red font show the interactors of GRFs.

To characterize the possible interaction networks of cassava GRFs, detailed analysis of GRFs from *Arabidopsis* and cassava were performed. Firstly, we applied STRING database [51] to construct the interaction networks of GRFs in *Arabidopsis*. We found that 13 GRFs were involved in the interaction network and these GRFs interacted with 18 target proteins, including *O*-methyltransferase, H^+^-ATPases, RING/FYVE/PHD zinc finger-containing protein, protein kinases, protein phosphatase 2C, and ascorbate peroxidase (Figure 10; Table S5). Secondly, the homologs of these proteins involved in the interaction network were identified from cassava with reciprocal best BLASTP analysis (Figure 10; Table S6). Thus, the potential interaction networks of GRFs were constructed in cassava. These results would lay a foundation for further investigation of GRF functions in cassava.

## 4. Discussion

Due to the regulatory role of GRFs in plant growth, development and response to abiotic stress, and the limited information for this gene family in cassava, it is essential to study the possible role of GRF genes in cassava. Here, we identified 15 GRFs from the cassava genome, which is expanded in comparison to GRFs from rice, *Arabidopsis*, common bean, tomato, *P. trichocarpa*, and *B. distachyon* [2,9,10,11,12,15,16,17,19]. Phylogenetic analysis showed that cassava GRFs were classified into the ε group and the non-ε group (Figure 1). This is consistent with previous classification of GRFs from *Arabidopsis*, rice, common bean, etc. [2,9,15]. Exon-intron organization analysis suggested that the ε group MeGRFs had more exons and introns than the non-ε group (Figure 3). This phenomenon is also observed in *Arabidopsis*, rice, common bean, *M. truncatula*, and *B. rapa* [2,9,15,18,53]. Besides, the exon number of GRFs is conserved among cassava (4–7), *Arabidopsis* (3–6), and rice (4–7). Conserved motif analysis showed that at least six 14–3–3 protein motifs existed in both ε group and non-ε group of cassava GRFs, indicating their conservation of protein sequences (Figure 2). Together, these evidences support the classification and conservation of cassava GRF family.

Accumulated evidences have revealed the important functions of 14–3–3 proteins in plant growth and development [20,21,22,23,24,25]. Investigation of the tissue expression patterns of 14–3–3 proteins would provide some clues on tissue development. In this study, we found that *MeGRF-6*, *-8*, *-10,* and *-15* in the ε group and *MeGRF-1*, *-4*, *-5*, *-7*, *-11*, *-12*, and *-13* in the non-ε group exhibited constitutive high expression levels (FPKM > 29) in leaves, stems, and storage roots of W14 and Arg7 (Figure 4). In *Arabidopsis*, *AtGF10* from the ε group, and *AtGF4* and *AtGF6* from the non-ε group showed abundant expression in shoots and roots [3]. Their homologous genes *MeGRF15* (homologs of *AtGF10*), *MeGRF5* (homologs of *AtGF4*), and *MeGRF-11*, *-12*, *-13* (homologs of *AtGF6*) in cassava also had high expression (Figure 1; Figure 4). In *B. distachyon*, seven out of 8 eight*GRF* genes showed low expression in roots, whereas high expression in stems, leaves, and spikelets [19]. In banana, five out of 25 *GRF* genes had constitutive high expression in roots, leaves, and fruits of two varieties [6]. In *M. truncatula*, eight *GRF* genes exhibited lower expression in leaves compared with in other tissues of roots, shoots, and flowers [53]. In common bean, most of the *GRFs* displayed high expression in flowers and stems, while low expression in pods and leaves [15]. In mesohexaploid *B. rapa*, most of the *GRF* genes showed low expression abundance in various tissues of roots, stems, leaves, and siliques [18]. Collectively, these studies revealed the tissue expression diversity of *GRFs* in various plant species. Compared with *GRFs* in other plants, the great number of *GRFs* with constitutive high expression in cassava indicates their important function in cassava development.

Biochemical and genetic evidences also confirmed the regulatory role of *GRFs* in plants response to abiotic stress and hormones [19,26,27,28,29,54,55]. To better understand cassava *GRFs* mediated transcriptional responses under abiotic stress and related signaling, *MeGRFs* expression were examined under various treatments. The results showed that *MeGRFs* could widely respond to cold, osmotic, salt, ABA, and H_2_O_2_ treatments at transcriptional levels (almost half members showing induction and half members showing repression under each treatment), suggesting their potential role in abiotic stress response (Figure 5, Figure 6, Figure 7, Figure 8 and Figure 9). The significant changes of *GRFs* at the transcriptional level under abiotic stress were also observed in other plants. In banana, nine, 13, and twelve *MaGRF* genes showed induction after cold, salt, and osmotic treatments, respectively, whereas 10, six, and seven *MaGRF* genes showed repression under the corresponding treatments [6]. In *B. distachyon*, *GRF* genes could be transcriptionally induced or repressed after osmotic, salt, ABA, and H_2_O_2_ treatments [19]. In common bean, all the identified *GRFs* were upregulated after cold treatment, and were induced or repressed upon drought and salt treatments [15]. In mesohexaploid *B. rapa*, most of the *GRFs* showed upregulation after salt, ABA, or SA treatments, but downregulation after dehydration or heat treatments [18]. These evidences are in accord with our expression analysis of cassava *GRFs*, further supporting the possible role of *MeGRFs* in abiotic stress responses.

Notably, *MeGRF3*, *MeGRF5*, *MeGRF6*, and *MeGRF11* were commonly upregulated by salt, osmotic, and ABA treatments; *MeGRF4* was commonly upregulated by cold, salt, ABA, and H_2_O_2_ treatments; and *MeGRF10* was commonly upregulated by cold, osmotic, and H_2_O_2_ treatments (Figure 5, Figure 6, Figure 7, Figure 8 and Figure 9). Numerous evidences indicated the positive role of *GRFs* in plants response to abiotic stress through affecting ABA pathway, stomatal behavior, ROS balance, and ions transports [19,26,27,28,29]. Thus, these cassava *GRFs* may be the converging points when cassava responds to abiotic stress, ABA signaling and H_2_O_2_, and can serve as candidates for genetic improvement of crop tolerance to abiotic stress.

As an important regulatory factor, GRFs elaborate their function through interacting with different clients. There is a need to investigate GRF mediated interaction network. In this study, we predicted 18 possible targets of 13 GRFs in cassava, including enzymes, transporters, protein kinases, and transcription factors (Figure 10; Table S6). These interactions have been confirmed in *Arabidopsis* [2,4,5,6,7]. The interaction relationship between GRFs and their targets, and the expression of cassava GRFs in each interaction group were shown in Table S7. This provided some clues for investigating the expression and function of GRFs and their targets. Further experimental validations would deepen the understanding of GRF functions in cassava.

In conclusion, this study identified 15 GRFs from cassava and investigated their phylogenetic classification, protein motif, and gene structure. Transcriptomic analysis showed the constitutively expressed or tissue specifically expressed *MeGRFs*. Expression analysis revealed the involvement of *MeGRFs* under abiotic stress and related signaling and identified some important candidates for improving crop resistances to multiple stresses. Furthermore, the GRF mediated interaction network was characterized, which would facilitate further study of their function in cassava. This systematic study will advance the understanding of GRF-mediated signal cascades in regulating cassava development and abiotic stress response, thereby supplying candidates for crop breeding.

## Supplementary Materials

The following are available online at http://www.mdpi.com/2073-4425/9/2/110/s1. Table S1: The primer sequences used for qRT-PCR. Table S2: Characteristics of GRFs in cassava. Table S3: The accession number of GRFs in *Arabidopsis* and rice. Table S4: Expression data of cassava *GRFs* in different tissues of W14 and Arg7. Table S5: The protein interaction relationship in the GRF-mediated interaction network in *Arabidopsis*. Table S6: The homologous genes in cassava from the interaction network. Table S7. The protein interaction relationship in the GRF-mediated interaction network and the expression of *GRFs* in cassava.

## References

[1] Sehnke P.C., DeLille J.M., Ferl R.J. (2002). Consummating signal transduction: the role of 14–3–3 proteins in the completion of signal-induced transitions in protein activity. Plant Cell.

[2] Chevalier D., Morris E.R., Walker J.C. (2009). 14–3–3 and FHA domains mediate phosphoprotein interactions. Annu. Rev. Plant Biol..

[3] Paul A.L., Denison F.C., Schultz E.R., Zupanska A.K., Ferl R.J. (2012). 14–3–3 phosphoprotein interaction networks—Does isoform diversity present functional interaction specification?. Front. Plant. Sci.

[4] Cotelle V., Leonhardt N. (2016). 14–3–3 Proteins in Guard Cell Signaling. Front. Plant. Sci.

[5] De Boer A.H., van Kleeff P.J., Gao J. (2013). Plant 14–3–3 proteins as spiders in a web of phosphorylation. Protoplasma.

[6] Li M., Ren L., Xu B., Yang X., Xia Q., He P., Xiao S., Guo A., Hu W., Jin Z. (2016). Genome-Wide Identification, Phylogeny, and Expression Analyses of the 14–3–3 Family Reveal Their Involvement in the Development, Ripening, and Abiotic Stress Response in Banana. Front. Plant. Sci..

[7] Saponaro A., Porro A., Chaves-Sanjuan A., Nardini M., Rauh O., Thiel G., Moroni A. (2017). Fusicoccin Activates KAT1 Channels by Stabilizing Their Interaction with 14–3–3 Proteins. Plant. Cell..

[8] De Vetten N.C., Lu G., Feri R.J. (1992). A maize protein associated with the G-box binding complex has homology to brain regulatory proteins. Plant Cell.

[9] Chen F., Li Q., Sun L., He Z. (2006). The rice 14–3–3 gene family and its involvement in responses to biotic and abiotic stress. DNA Res..

[10] Yao Y., Du Y., Jiang L., Liu J.Y. (2007). Molecular analysis and expression patterns of the 14–3–3 gene family from *Oryza sativa*. J. Biochem. Mol. Biol..

[11] DeLille J.M., Sehnke P.C., Ferl R.J. (2001). The *Arabidopsis* 14–3–3 family of signaling regulators. Plant. Physiol..

[12] Rosenquist M., Alsterfjord M., Larsson C., Sommarin M. (2001). Data mining the *Arabidopsis* genome reveals fifteen 14–3–3 genes. Expression is demonstrated for two out of five novel genes. Plant. Physiol..

[13] Li X., Dhaubhadel S. (2011). Soybean 14–3–3 gene family: Identification and molecular characterization. Planta.

[14] Sun G., Xie F., Zhang B. (2011). Transcriptome-wide identification and stress properties of the 14–3–3 gene family in cotton (*Gossypium hirsutum* L.). Funct. Integr. Genom..

[15] Li R., Jiang X., Jin D., Dhaubhadel S., Bian S., Li X. (2015). Identification of 14–3–3 Family in Common Bean and Their Response to Abiotic Stress. PLoS ONE.

[16] Xu W.F., Shi W.M. (2006). Expression profiling of the 14–3–3 gene family in response to salt stress and potassium and iron deficiencies in young tomato (*Solanum lycopersicum*) roots: analysis by real-time RT-PCR. Ann. Bot.

[17] Tian F., Wang T., Xie Y., Zhang J., Hu J. (2015). Genome-wide identification, classification, and expression analysis of 14–3–3 gene family in *Populus*. PLoS ONE.

[18] Chandna R., Augustine R., Kanchupati P., Kumar R., Kumar P., Arya G.C., Bisht N.C. (2016). Class-Specific Evolution and Transcriptional Differentiation of 14–3–3 Family Members in Mesohexaploid *Brassica rapa*. Front. Plant. Sci..

[19] He Y., Zhang Y., Chen L., Wu C., Luo Q., Zhang F., Wei Q., Li K., Chang J., Yang G. (2017). Member of the 14-3–3 Gene Family in *Brachypodium distachyon*, *BdGF14d*, Confers Salt Tolerance in Transgenic Tobacco Plants. Front. Plant. Sci..

[20] Keicher J., Jaspert N., Weckermann K., Moller C., Throm C., Kintzi A., Oecking C. (2017). *Arabidopsis* 14–3–3 ε members contribute to polarity of PIN auxin carrier and auxin transport-related development. Elife.

[21] Vercruyssen L., Tognetti V.B., Gonzalez N., Van Dingenen J., De Milde L., Bielach A., De Rycke R., Van Breusegem F., Inze D. (2015). GROWTH REGULATING FACTOR5 stimulates *Arabidopsis* chloroplast division, photosynthesis, and leaf longevity. Plant. Physiol..

[22] Van Kleeff P.J., Jaspert N., Li K.W., Rauch S., Oecking C., de Boer A.H. (2014). Higher order *Arabidopsis* 14–3–3 mutants show 14–3–3 involvement in primary root growth both under control and abiotic stress conditions. J. Exp. Bot..

[23] He Y., Wu J., Lv B., Li J., Gao Z., Xu W., Baluska F., Shi W., Shaw P.C., Zhang J. (2015). Involvement of 14–3–3 protein GRF9 in root growth and response under polyethylene glycol-induced water stress. J. Exp. Bot..

[24] Sun X., Luo X., Sun M., Chen C., Ding X., Wang X., Yang S., Yu Q., Jia B., Ji W. (2014). Glycine soja 14–3–3 protein GsGF14o participates in stomatal and root hair development and drought tolerance in *Arabidopsis thaliana*. Plant. Cell. Physiol..

[25] Zhou Y., Zhang Z.T., Li M., Wei X.Z., Li X.J., Li B.Y., Li X.B. (2015). Cotton (*Gossypium hirsutum*) 14–3–3 proteins participate in regulation of fibre initiation and elongation by modulating brassinosteroid signalling. Plant Biotechnol. J..

[26] Chen Y., Zhou X., Chang S., Chu Z., Wang H., Han S., Wang Y. (2017). Calcium-dependent protein kinase 21 phosphorylates 14–3–3 proteins in response to ABA signaling and salt stress in rice. Biochem. Biophys. Res. Commun..

[27] Kaundal A., Ramu V.S., Oh S., Lee S., Pant B., Lee H.K., Rojas C.M., Senthil-Kumar M., Mysore K.S. (2017). GENERAL CONTROL NONREPRESSIBLE4 Degrades 14–3–3 and the RIN4 Complex to Regulate Stomatal Aperture with Implications on Nonhost Disease Resistance and Drought Tolerance. Plant Cell.

[28] Yang L., You J., Wang Y., Li J., Quan W., Yin M., Wang Q., Chan Z. (2017). Systematic analysis of the G-box Factor 14–3–3 gene family and functional characterization of GF14a in *Brachypodium distachyon*. Plant Physiol. Biochem..

[29] Liu Z., Jia Y., Ding Y., Shi Y., Li Z., Guo Y., Gong Z., Yang S. (2017). Plasma Membrane CRPK1-Mediated Phosphorylation of 14–3–3 Proteins Induces Their Nuclear Import to Fine-Tune CBF Signaling during Cold Response. Mol. Cell.

[30] Zidenga T., Leyva-Guerrero E., Moon H., Siritunga D., Sayre R. (2012). Extending Cassava root shelf life via reduction of reactive oxygen species production. Plant Physiol..

[31] Zhang P., Wang W.Q., Zhang G.L., Kaminek M., Dobrev P., Xu J., Gruissem W. (2010). Senescence-inducible expression of isopentenyl transferase extends leaf life, increases drought stress resistance and alters cytokinin metabolism in Cassava. J. Integr. Plant Biol..

[32] Xu J., Duan X., Yang J., Beeching J.R., Zhang P. (2013). Enhanced reactive oxygen species scavenging by overproduction of superoxide dismutase and catalase delays postharvest physiological deterioration of Cassava storage roots. Plant Physiol..

[33] Kawahara Y., de la Bastide M., Hamilton J.P., Kanamori H., McCombie W.R., Ouyang S., Schwartz D.C., Tanaka T., Wu J., Zhou S. (2013). Improvement of the *Oryza sativa* Nipponbare reference genome using next generation sequence and optical map data. Rice.

[34] The UniProt Consortium (2015). UniProt: A hub for protein information. Nucleic Acids Res..

[35] Prochnik S., Marri P.R., Desany B., Rabinowicz P.D., Kodira C., Mohiuddin M., Rodriguez F., Fauquet C., Tohme J., Harkins T. (2012). The *Cassava* Genome: Current Progress, Future Directions. Trop. Plant Biol..

[36] Eddy S.R. (2011). Accelerated Profile HMM Searches. PLoS Comput. Biol..

[37] Finn R.D., Clements J., Eddy S.R. (2011). HMMER web server: Interactive sequence similarity searching. Nucleic Acids Res..

[38] Altschul S.F., Gish W., Miller W., Myers E.W., Lipman D.J. (1990). Basic local alignment search tool. J. Mol. Biol..

[39] Finn R.D., Coggill P., Eberhardt R.Y., Eddy S.R., Mistry J., Mitchell A.L., Potter S.C., Punta M., Qureshi M., Sangrador-Vegas A. (2016). The Pfam protein families database: Towards a more sustainable future. Nucleic Acids Res..

[40] Marchler-Bauer A., Derbyshire M.K., Gonzales N.R., Lu S.N., Chitsaz F., Geer L.Y., Geer R.C., He J., Gwadz M., Hurwitz D.I. (2015). CDD: NCBI’s conserved domain database. Nucleic Acids Res..

[41] Larkin M.A., Blackshields G., Brown N.P., Chenna R., McGettigan P.A., McWilliam H., Valentin F., Wallace I.M., Wilm A., Lopez R. (2007). Clustal W and Clustal X version 2.0. Bioinformatics.

[42] Tamura K., Peterson D., Peterson N., Stecher G., Nei M., Kumar S. (2011). MEGA5: Molecular evolutionary genetics analysis using maximum likelihood, evolutionary distance, and maximum parsimony methods. Mol. Biol. Evol..

[43] Hannon Lab. FASTX-Toolkit. http://hannonlab.cshl.edu/fastx_toolkit.

[44] Braham Bioinformatics FastQC. http://www.bioinformatics.babraham.ac.uk/projects/fastqc.

[45] John Hopkins University. Center for Computational biology (CCB) TopHat. http://ccb.jhu.edu/software/tophat/index.shtml/.

[46] Trapnell C., Roberts A., Goff L., Pertea G., Kim D., Kelley D.R., Pimentel H., Salzberg S.L., Rinn J.L., Pachter L. (2012). Differential gene and transcript expression analysis of RNA-seq experiments with TopHat and Cufflinks. Nat. Protoc..

[47] Gasteiger E., Gattiker A., Hoogland C., Ivanyi I., Appel R.D., Bairoch A. (2003). ExPASy: The proteomics server for in-depth protein knowledge and analysis. Nucleic Acids Res..

[48] Brown P., Baxter L., Hickman R., Beynon J., Moore J.D., Ott S. (2013). MEME-LaB: Motif analysis in clusters. Bioinformatics.

[49] Mulder N., Apweiler R. (2007). InterPro and InterProScan: Tools for protein sequence classification and comparison. Methods Mol. Biol..

[50] Hu B., Jin J., Guo A.Y., Zhang H., Luo J., Gao G. (2015). GSDS 2.0: An upgraded gene feature visualization server. Bioinformatics.

[51] Szklarczyk D., Franceschini A., Wyder S., Forslund K., Heller D., Huerta-Cepas J., Simonovic M., Roth A., Santos A., Tsafou K.P. (2015). STRING v10: Protein-protein interaction networks, integrated over the tree of life. Nucleic Acids Res..

[52] Livak K.J., Schmittgen T.D. (2001). Analysis of relative gene expression data using real-time quantitative PCR and the 2^−ΔΔC^T Method. Methods.

[53] Qin C., Cheng L., Shen J., Zhang Y., Cao H., Lu D., Shen C. (2016). Genome-Wide Identification and Expression Analysis of the 14–3–3 Family Genes in *Medicago truncatula*. Front. Plant Sci..

[54] Wang H., Yang C., Zhang C., Wang N., Lu D., Wang J., Zhang S., Wang Z.X., Ma H., Wang X. (2011). Dual role of BKI1 and 14–3–3 s in brassinosteroid signaling to link receptor with transcription factors. Dev. Cell.

[55] Gampala S.S., Kim T.W., He J.X., Tang W., Deng Z., Bai M.Y., Guan S., Lalonde S., Sun Y., Gendron J.M. (2007). An essential role for 14–3–3 proteins in brassinosteroid signal transduction in *Arabidopsis*. Dev. Cell.

